# Neurite outgrowth on a fibronectin isoform expressed during peripheral nerve regeneration is mediated by the interaction of paxillin with α4β1 integrins

**DOI:** 10.1186/1471-2202-8-44

**Published:** 2007-06-29

**Authors:** Mariette Vogelezang, Ulrike B Forster, Jaewon Han, Mark H Ginsberg, Charles ffrench-Constant

**Affiliations:** 1 Dept of Pathology, University of Cambridge, Tennis Court Road, Cambridge, CB2 1QP, UK and Cambridge Centre for Brain Repair, University of Cambridge, The E.D Adrian Building, Forvie Site, Robinson Way, Cambridge, CB2 2PY, UK; 2 Dept of Medicine, University of California San Diego, 9500 Gilman Drive, La Jolla, CA 92093-0726, USA; 3 Center for Cancer Research, Massachusetts Institute of Technology, 77 Massachusetts Avenue, E17-227, Cambridge, MA 01235-4307, USA

## Abstract

**Background:**

The regeneration of peripheral nerve is associated with a change in the alternative splicing of the fibronectin primary gene transcript to re-express embryonic isoforms containing a binding site for α4β1 integrins that promote neurite outgrowth. Here we use PC12 cells to examine the role of the interaction between paxillin and the α4 integrin cytoplasmic domain in neurite outgrowth.

**Results:**

Expression of α4 with mutations in the paxillin-binding domain reduced neurite outgrowth on recombinant embryonic fibronectin fragments relative to wild type α4. Over-expression of paxillin promoted neurite outgrowth while a mutant isoform lacking the LD4 domain implicated in the regulation of ARF and Rac GTPases was less effective. Optimal α4-mediated migration in leucocytes requires spatial regulation of α4 phosphorylation at Ser^988^, a post-translational modification that blocks paxillin binding to the integrin cytoplasmic domain. In keeping with this α4(S988D), which mimics phosphorylated α4, did not promote neurite outgrowth. However, α4 was not phosphorylated in the PC12 cells, and a non-phosphorylatable α4(S988A) mutant promoted neurite outgrowth indistinguishably from the wild type integrin.

**Conclusion:**

We establish the importance of the α4 integrin-paxillin interaction in a model of axonal regeneration and highlight differing dependence on phosphorylation of α4 for extension of neuronal growth cones and migration of non-neural cells.

## Background

Regrowth of the peripheral nervous system (PNS) after injuries such as axotomy is associated with changes in the expression of extracellular matrix (ECM) molecules in the environment traversed by the regenerating growth cones [1]. These changes may promote regeneration in at least two different ways. First, by generating increased levels of molecules such as laminin (Ln) and fibronectin (Fn) that can promote neurite outgrowth in cell culture and are therefore likely to promote regeneration in vivo [2,3]. Second, by altering the pattern of alternative splicing so as to switch from isoforms of ECM proteins expressed in adult nerve to those expressed earlier during a time of axon growth in development. An example of the second mechanism is provided by Fn, whose primary gene transcript is alternatively spliced in three regions [4-9]. Two of these include or exclude single Fn type III repeats (EIIIA and EIIIB) while the third partially or completely excludes a region (V or IIICS) that contains a cell binding sequence Leu-Asp-Val (LDV) recognised by the integrin α4β1 [10,11]. While these regions are excluded from adult Fns in a cell and tissue specific pattern [12-14], we have previously found that sciatic nerve injury and regeneration in the adult rat is associated with a change in the pattern of alternative splicing and the re-expression of embryonic Fns that include all these regions [15,16].

Recombinant Fns that include the entire V region promote neurite outgrowth from dorsal root ganglion (DRG) neurons or PC12 cells (a cell line model of PNS neurones) more effectively than those containing only the RGD sequence present in the 10th type III repeat present in all Fns [17]. As the α4 integrin subunit is expressed on regenerating growth cones *in vivo*, we have therefore proposed that the re-expression of embryonic Fns following injury contributes to regeneration of the adult PNS by enhancing α4β1 integrin signalling [17]. The identification of α4 integrins as part of the regenerative mechanism in the PNS is of particular interest as these integrins play an important role in cell migration in other cell types [18,19]. The adaptor protein, paxillin, which binds directly to the α4 cytoplasmic domain, is an important downstream signalling molecule in this process [20]. Transgenic knock-in mice expressing only α4 integrin with a Y991A mutation within the paxillin binding site that blocks the interaction show impaired leucocyte recruitment during inflammation [21]. A physiological modification that blocks binding of paxillin is generated by phosphorylation of the α4 integrin cytoplasmic domain, detected in certain T cell lines using an antiserum raised against a synthetic peptide containing phosphoserine at Ser^988 ^[22]. Phosphorylation is localised to the leading edge of migrating CHO cells [23], and the reversibility of paxillin binding enabled by phosphorylation and dephosphorylation of α4 may be important for migration, as both S988A (that inhibits phosphorylation) and S988D (that mimics phosphorylation) mutations inhibit migration in cell culture [24]. We have shown previously that paxillin is also associated specifically with α4 in DRG neurons and PC12 cells [17]. This suggests the hypothesis that paxillin also makes an essential contribution to α4-mediated growth cone motility and PNS regeneration by virtue of the association with the integrin cytoplasmic domain. Here we test this hypothesis by examining the requirement for the α4-paxillin interaction in neurite outgrowth and find that the interaction is required; however, in contrast to non-neuronal cell migration, α4 phosphorylation is dispensable for neurite outgrowth.

## Results

**Figure 1:**
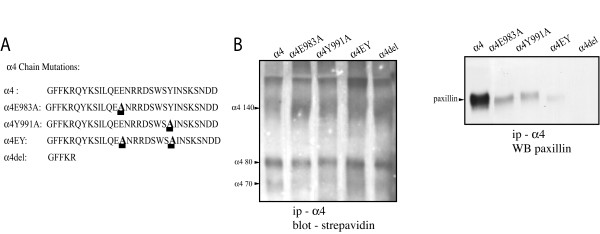
Mutations in the paxillin-binding region of the α4 cytoplasmic domain reduce paxillin binding A) The amino acid sequence of the cytoplasmic domains of the α4 integrins expressed in PC12 cells, starting at the conserved juxtamembrane GFFKR sequence. In all cases, the transmembrane and extracellular domains were wild-type human α4 integrin. The different mutations in the paxillin binding domain are underlined, and the cytoplasmic domain deletion (α4del) is also shown. B) The left panel shows a streptavidin peroxidase/ECL blot of 1% Triton X-100 lysates of biotin-labelled PC12 cells expressing the different α4 constructs following immunoprecipitation with anti-human α4 extracellular domain antibodies and SDS-PAGE on 7% gels. Note the similar expression of the different mutants. The right panel then shows the same immunoprecipitates western blotted with biotin-labelled anti-paxillin antibodies so as to examine paxillin binding to each integrin cytoplasmic domain in the PC12 cells. Note that the single amino acid mutations (α4E983A and α4Y991A) reduce binding, the double mutation (α4EY) even more so, and that no binding is seen following deletion of the α4 cytoplasmic domain (α4del).

To examine α4-mediated neurite outgrowth we used PC12 cells as model neurons. These cells do not express α4 integrin and transfection with α4 enables them to manifest neurite outgrowth on Fn isoforms that contain the α4 integrin binding site [17]. To assess the requirement for paxillin binding to the α4 cytoplasmic domain for this neurite outgrowth, we first expressed α4 subunits into which alanine substitutions had been inserted into either or both of two amino acids essential for paxillin binding; Glu^983 ^and Tyr^991 ^[25]. As controls we expressed either full-length α4 or α4 lacking the cytoplasmic domain sequences C-terminal to the GFFKR motif (Fig 1A). To demonstrate the expected effects of these mutations on paxillin binding, we immunoprecipitated lysates of the PC12 cells with antibodies against the extracellular domain of α4 and then western blotted the protein complexes pulled down with anti-paxillin antibodies. As shown in Fig 1B, the α4 subunits were all expressed in the PC12 cells but bound paxillin to differing degrees; while full-length α4 complexed with high levels of paxillin, α4 with either single mutation bound much lower levels and α4 with the double mutation showed virtually no binding. As expected, the α4 subunit lacking the majority of the cytoplasmic domain did not bind paxillin, illustrating the specificity of the assay.

**Figure 2:**
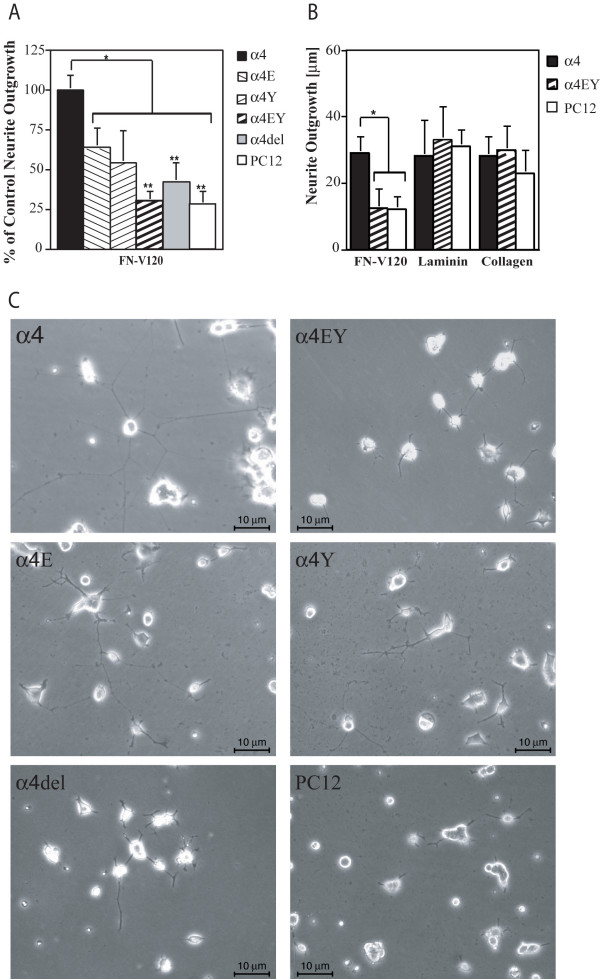
Mutations in the paxillin-binding region of the α4 cytoplasmic domain inhibit α4-mediated neurite outgrowth. A) Neurite outgrowth from PC12 cells expressing the different α4 constructs after 24 hrs plated onto recombinant Fn fragments containing the V120 sequence (Fn V120, 50 nM) within which the α4-binding LDV motif is present. In each experiments the longest neurites in at least 100 cells were measured and the results normalised to the median values seen in cells expressing wild type α4. The data shown are the mean ± SEM of 6 experiments. Note the reduction in the single mutants (* = P < 0.01), and complete loss of any α4-mediated outgrowth with the double mutant and cytoplasmic domain deletion (** = P < 0.005), with levels now the same as those seen on mock-transfected PC12 cells. B) Neurite outgrowth from mock-transfected PC12 cells and those expressing wild-type α4 or the double mutant. Note that, as in A), outgrowth on Fn V120 requires a normal α4 cytoplasmic domain while outgrowth on laminin and collagen does not and is similar in all cell lines. C) Representative examples of neurite outgrowth in PC12 cells expressing the different α4 constructs.

To examine the effects of these mutations on α4-mediated neurite outgrowth, we then grew these PC12 cells on recombinant Fn fragments containing the V120 region for 24 hrs. The extent of neurite outgrowth correlated with the degree of paxillin binding to the α4 cytoplasmic domain (Fig 2A). Either single mutation reduced neurite outgrowth while the double mutation resulted in a complete loss of α4-mediated neurite outgrowth, with the degree of outgrowth now being identical to the low level seen with wild type PC12 cells or with PC12 cells expressing the α4 subunit lacking the majority of the cytoplasmic domain. To ask whether this effect of perturbing paxillin interactions was specific for α4-mediated neurite outgrowth, we repeated the experiments with cells expressing wild type α4 or the double mutation on laminin, collagen or Fn V120. Laminin and collagen bind to different integrins on the cell surface (α1β1, α3β1, α6β1 and α7β1 for laminin, α1β1 and α2β1 for collagen [26]) and promote neurite outgrowth in PC12 cells [27,28]. Expression of α4, either wild-type or mutant, had no effect on this neurite outgrowth while, as before, only the cells expressing wild-type α4 showed enhanced neurite outgrowth on Fn V120 (Fig 2B) confirming that the loss of the paxillin association with α4 only perturbs α4-mediated neurite outgrowth.

**Figure 3:**
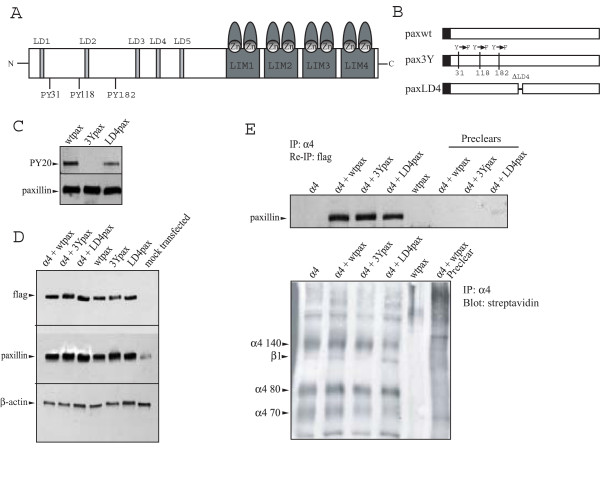
Expression and binding to α4 of paxillin mutant isoforms. A) Schematic representation of paxillin, showing the position of the 3 phosphorylated tyrosine residues, the LD domains and the LIM domains. B) The three constructs used to examine paxillin function; wild-type paxillin (paxwt), Tyr to Phe mutations of the 3 tyrosines (pax3Y) and an LD4 domain deletion (paxLD4). All three contain a FLAG tag. C) Western blotting of lysates from PC12 cells expressing the different paxillin constructs. All three are expressed at a similar level, but the pax3Y mutant is not recognised by the anti-phosphotyrosine PY20 antibody. D) Western blots using anti-FLAG tag and anti-paxillin antibodies of lysates from PC12 cells expressing the different paxillin constructs with or without co-expression of wild-type α4 integrin, and from mock-transfected PC12 cells. The anti-FLAG (top panel) recognises only expressed paxillin, while the anti-paxillin antibody (middle panel) recognises both expressed and endogenous paxillin. Note that the normal endogenous levels of paxillin (seen in the mock-transfected cells with the anti-paxillin antibody) are lower than those seen following expression of the three constructs, with or without α4 (middle panel), and that all cell lines contain similar levels of expressed paxillin (top panel). A β-actin loading control is also shown in the lower panel. E) Lysates of biotin-labelled PC12 cells expressing either wild-type α4 alone, wild-type paxillin alone or wild-type α4 with the different paxillin constructs immunoprecipitated with anti-α4 as in Fig 1 and then re-immunoprecipitated (Re-IP) with anti-FLAG antibodies before western blotting with anti-paxillin antibody (top panel) or visualised by streptavidin-peroxidase/ECL (lower panel). All four cell lines transfected with α4 constructs express the integrin at similar levels (lower panel), and all expressed (wild-type and mutant) paxillins bind to the integrin equally well (upper panel). In the absence of any α4 integrin (wtpax only in upper panel), no expressed paxillin is detected, confirming the specificity of the assay. Note that the anti-FLAG antibody used to detect paxillin would not be expected to detect the endogenous paxillin associated with α4, and so no band is seen in the α4-only lane of the upper panel.

To show directly that paxillin promotes neurite outgrowth in response to Fn V120 substrates, we over-expressed wild-type or mutant paxillins in PC12 cells also expressing wild-type α4. The structure of wild-type paxillin is shown in Fig 3A, and the two different paxillin mutants used in the study are shown in Fig. 3B. One lacks the LD4 domain that promotes neurite outgrowth in PC12 cells grown on collagen substrates [28]. The other has Tyr to Phe substitutions at Tyr^31^, Tyr^118 ^and Tyr^182^. This prevents the phosphorylation of Tyr^31 ^and Tyr^118 ^implicated in cell motility [29] but has no effect on neurite outgrowth on collagen [28]. Western blot experiments confirmed that tyrosine phosphorylation was not observed in the triple mutant paxillin, whilst the wild type and LD4 deletion mutant forms were recognised by the anti-phosphotyrosine antibody (Fig 3C). To determine the relative expression levels of expressed and endogenous paxillin, we performed western blots using antibodies against paxillin (that will recognise both the endogenous and expressed molecules) and against the FLAG-tag present only in the expressed form of paxillin. As shown in Fig 3D, there were greater levels of the expressed paxillins than the endogenous paxillin, and the levels of the mutant paxillins were comparable with those seen with the wild-type construct (Fig 3D). To confirm that the expressed paxillins associated with α4, we western blotted using anti-paxillin antibodies cell lysates immunoprecipitated first with anti-α4 and then with the anti FLAG-tag antibody that detects only the expressed paxillin. All the expressed paxillins associated with α4 (Fig 3E). Notably the LD4 deletion mutant associated with the integrin, even though the critical residues for α4 binding have been shown to be present in an Ala^176 ^to Asp^275 ^fragment containing the LD3 and LD4 domains [30].

**Figure 4:**
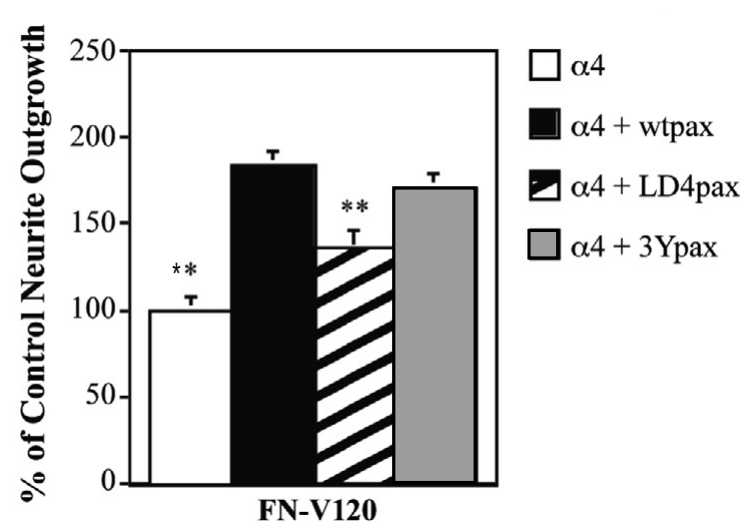
The LD4 domain of paxillin contributes to α4-mediated neurite outgrowth. Neurite outgrowth of PC12 cells expressing α4 and the different paxillin constructs, measured as in Fig. 1. The data shown are the mean ± SEM of 3 experiments. Note that paxillin over-expression enhances neurite outgrowth, but the paxillin lacking the LD4 domain (LD4pax) does so less effectively than wild-type paxillin (wtpax) or paxillin with Tyr to Phe mutations of the 3 tyrosines (3Ypax) (** = P < 0.05 as compared to the level of outgrowth in cells expressing α4 and wtpax).

Next, we examined neurite outgrowth in PC12 cells co-expressing wild-type α4 and the different paxillins. In cells expressing wild-type paxillin, we found enhanced neurite outgrowth on Fn V120 compared with cells expressing α4 alone (Fig 4). In keeping with the previous studies [28], no significant difference in the extent of neurite outgrowth on Fn V120 substrates was seen between PC12 cells expressing wild type and the triple tyrosine mutant paxillin, while the LD4 domain mutant supported neurite outgrowth to a significantly reduced extent (Fig 4). These over-expression studies therefore confirm a role for paxillin in promoting neurite outgrowth on Fn V120 substrates. Taken together with the studies above showing that association of paxillin with the integrin cytoplasmic domain is required for outgrowth, they show that the recruitment of paxillin by α4 integrin represents an important mechanism for neurite outgrowth on these embryonic fibronectins.

**Figure 5:**
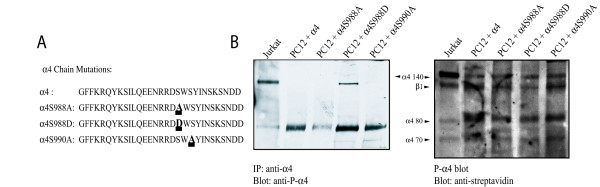
Mutations of Ser^988 ^in the α4 cytoplasmic domain mimic phosphorylation. A) The amino acid sequence of the cytoplasmic domains of the α4 integrins expressed in PC12 cells, starting at the conserved juxtamembrane GFFKR sequence. As in Fig 1, the transmembrane and extracellular domains were wild-type human α4 integrin. The different mutations are underlined. B) The left panel shows an anti-phospho Ser988 α4 integrin blot of 1% Triton X-100 lysates of biotin-labelled PC12 cells expressing the different α4 constructs following immunoprecipitation with anti-human α4 extracellular domain antibodies and SDS-PAGE on 7% gels. Note that α4 integrins with the phospho-mimicking mutation S988D are labelled, while those with the S988A and control S990A mutations are not. Jurkat T cells are shown as a positive control. The right hand panel then shows the same blot stripped and reprobed with streptavidin peroxidase/ECL to confirm the similar expression of the different α4 constructs.

**Figure 6:**
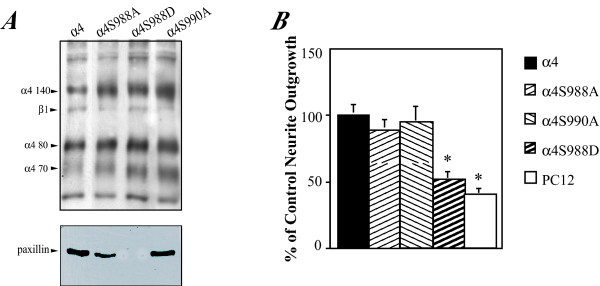
Mutations of Ser^988 ^in the α4 cytoplasmic domain that mimic phosphorylation reduce paxillin binding and inhibit α4-mediated neurite outgrowth. A) Lysates of biotin-labelled PC12 cells expressing the different α4 constructs immunoprecipitated with anti-α4 antibody as in Fig 1 and visualised by streptavidin peroxidase/ECL (upper panel) or by western blotting with anti-paxillin antibody (lower panel). Note that the phospho-mimicking S988D mutation prevents paxillin binding and co-immunoprecipitation. B) Neurite outgrowth of PC12 cells expressing the different α4 constructs and mock-transfected PC12 cells measured as in Fig. 1. The data shown are the mean ± SEM of 3 experiments. Note that the S988D mutation also inhibits α4-mediated neurite outgrowth (* = P < 0.01).

As outlined in the introduction, the binding of paxillin to the α4 integrin is inhibited by phosphorylation of Ser^988^. The temporal and spatial regulation of paxillin binding enabled by phosphorylation and dephosphorylation may have an important physiological role in cell motility, as both S988A (that inhibits phosphorylation) and S988D (that mimics phosphorylation) mutations inhibit migration in cell culture [24]. In the final part of this study, therefore, we examined the role of phosphorylation of Ser^988 ^in neurite outgrowth on Fn V120 substrates. We transfected PC12 cells with either wild type α4 or α4 with S988A or S988D mutations. As an additional control, we used a control S990A mutation that does not alter paxillin binding (Fig 5A). All four α4 constructs were equally expressed in PC12 cells (Fig 5B). Wild type α4 was not phosphorylated in PC12 cells as judged by immuno blotting with a phospho-α4 specific antibody whereas it was heavily phosphorylated in Jurkat cells (a T cell line) (Fig. 5B). In contrast, the α4(S988D) was labelled by this antibody in PC12 cells suggesting that this mutation is also phospho-mimetic with respect to recognition by this antibody. We confirmed the effect of these mutations on the binding of paxillin to the α4 cytoplasmic domain by immunoprecipitating α4 from lysates of cells expressing each of the four different α4 subunits and detecting paxillin by immunoblotting. As expected, no associated paxillin was seen with the phospho-mimicking S988D mutation, whilst all others bound paxillin as expected (Fig 6A). Lastly, we examined neurite outgrowth in PC12 cells expressing each of the four α4 subunits on Fn V120. As before, wild-type α4 promoted neurite outgrowth and this was not affected by the S988A or S990A mutations. In contrast, however, the S988D mutation that blocked paxillin binding showed no α4-mediated neurite outgrowth, as the levels of outgrowth were identical to those of PC12 cells not expressing α4 integrin (Fig 6B). We conclude that mutations that mimic phosphorylation of Ser^988 ^will prevent paxillin binding in PC12 cells, and therefore inhibit the neurite outgrowth promoted by α4 integrin on Fn V120 substrates. However, the lack of detectable phosphorylation of wild-type α4 in the PC12 cells and the efficacy of α4(S988A) in promoting neurite outgrowth suggests that the reversibility of paxillin binding enabled by phosphorylation of Ser^988 ^is not necessary for neurite outgrowth.

## Discussion

We have shown that the association of the adaptor protein, paxillin, with the α4 integrin cytoplasmic domain is required for α4-mediated neurite outgrowth in PC12 cells on embryonic Fns that are expressed during nerve regeneration. In the absence of this association, the enhanced neurite outgrowth on Fn V120 is lost. Conversely, overexpression of paxillin enhances α4-dependent neurite outgrowth on this substrate. As alternative splicing of the Fn primary gene transcript following PNS injury increases the number of Fns containing V120 in the regenerating nerve, and α4 is expressed on regenerating growth cones *in vivo *[16,17], we suggest that the α4-paxillin interaction may play a key role in PNS regeneration. The upregulation of different ECM molecules during PNS regeneration suggests that other integrins will also contribute to regeneration. Two candidates are α5β1, that is also expressed on regenerating growth cones in vivo [2], and α7β1, a laminin receptor expressed on a subset of DRG neurons and also shown to be required for normal regeneration of motor neurones in the facial nerve [31,32]. However, as α4 and the closely related α9 are the only integrin α subunits reported to bind paxillin directly [20,33], it is likely that these integrins will activate different and parallel downstream pathways so providing an enhanced regenerative response triggered by the change in Fn splicing.

The demonstration of a functional requirement for paxillin binding to a specific integrin to promote neurite outgrowth extends previous work on signalling within growth cones. Paxillin is localised within neuronal growth cones within dynamic focal adhesions that assemble at the leading edge, enlarge and finally disassemble while remaining in place as the growth cone advances [34]. It becomes tyrosine phosphorylated on neurite outgrowth [35,36]. Tyrosine phosphorylation of paxillin at Tyr^31 ^and Tyr^118 ^provide a binding site for the adaptor protein Crk [37,38]. Overexpression of Crk promotes neurite outgrowth in PC12 cells [39], and also promotes cell motility in a bladder cancer cell line [29]. Surprisingly, therefore, but in agreement with others [28], we find no evidence that inhibiting tyrosine phosphorylation by expression of a paxillin with Tyr to Phe mutations in these residues inhibits the ability of over-expressed paxillin to enhance neurite outgrowth. Two other regions of paxillin have been implicated in neurite outgrowth signalling. First, Ser ^85 ^(Ser ^83 ^in the rat) that is phosphorylated by p38 MAPK. This phosphorylation promotes outgrowth that can be inhibited by a Ser to Ala mutation in this position [40]. Second, the LD4 domain within the leucine-rich domains, that provides a binding site for a number of other signalling proteins [41,42] and deletion of which inhibits neurite outgrowth in PC12 cells growing on collagen substrates [28]. Our results show markedly less neurite outgrowth following expression of the LD4 deletion mutant than full-length paxillin, suggesting an important role of the LD4 domain in α4 integrin-mediated neurite formation.

At least three possible mechanisms by which the LD4 domain might promote neurite outgrowth have been described. First, by the recruitment of the focal adhesion kinases FAK and Pyk2, both of which interact directly with the LD4 domain [41], are co-immunoprecipitated with paxillin in PC12 cells [43] and are activated by interaction with the α4 cytoplasmic domain in T cells [44]. Activation of these kinases occurs during growth factor-induced neurite outgrowth in PC12 cells, and inhibition of their activity by expression of a C-terminal construct (PRNK and FRNK, which act as dominant negative isoforms) blocks this outgrowth [28]. Second, by recruitment of the Arf-GAP PKL. This in turn recruits the p21-associated kinase (PAK1) via a linking interaction with PIX [45], and PAK1 localisation to the membrane in PC12 cells has been shown to promote neurite outgrowth [46], potentially via the activation of cdc42 [42]. Third, by the regulation of the Rho GTPase Rac, as demonstrated by the prolonged levels of Rac activation, loss of directional migration and randomly-orientated lamellopodia formation in CHO cells expressing an LD4 deletion mutant of paxillin [47]. As expression of a constitutively active form of Rac in PC12 cells promotes cell spreading but not neurite outgrowth, whilst dominant negative Rac inhibits neurite outgrowth [46], precise regulation of Rac activity is clearly essential for neurite formation by PC12 cells.

Directional cell migration is associated with high levels of Rac activity at the leading edge of the cell [48]. A key question for future studies will therefore be the role of paxillin and α4 integrin in the spatial control of Rac activity within the growth cone. The phosphorylated α4 integrins found in T cell lines will not bind paxillin [22], and are localised to the leading edge of migrating CHO cells, while non-phosphorylated integrins are found in the lateral edges [23]. As the α4-paxillin interaction inhibits Rac activity and lamellopodia formation, the extension of cellular protrusions driven by Rac and required for movement are therefore restricted to the leading edge of the cell and directionality of migration is achieved [23,49]. A mechanism by which the paxillin-integrin interaction reduces Rac activity is provided by recent work showing that recruitment of the Arf-GAP GIT1 inactivates Arf activity, which in turn results in decreased Rac activation [49]. Integrin phosphorylation therefore plays a key role in directional migration in some non-neural cells by providing a mechanism for the spatial regulation of paxillin dissociation from the α4 cytoplasmic domain.

## Conclusion

Peripheral nerve repair is associated with a re-expression of embryonic isoforms of fibronectin that contain a binding site for the α4β1 integrin. This integrin is expressed on regenerating growth cones and promotes neurite outgrowth on embryonic fibronectins in cell culture. Using PC12 neuronal cells as a model system, we have shown here that the interaction of the adaptor protein paxillin with the integrin cytoplasmic domain is required for this outgrowth. We have also found that, in contrast to studies using T cells, there is no biochemical evidence for phosphorylation of α4 integrins in PC12 cells, suggesting that the dissociation of paxillin from the integrin mediated by this phosphorylation is either not required for growth cone motility or is achieved through different means. This points to potentially interesting differences between the mechanisms of two distinct forms of cell movement; directional migration in isolated cells and growth cone motility at the end of a cell process or axon.

## Methods

### Reagents

The following antibodies were used: mouse anti-human α4, clone HP2/1 (Chemicon); hamster anti-rat β1, clone HA2/5 (Pharmingen), mouse anti-paxillin, clone 349 (Transduction Laboratories), mouse anti-FLAG M2 (Sigma), anti-phosphotyrosine antibody PY20 (Transduction Laboratories). The rabbit phospho-specific anti-α1 antibody was generated against a synthetic peptide containing phospho serine at Ser^988 ^as previously described [22]. Recombinant Fn fragments encompassing repeats III-8-15 and containing all three possible combinations of the V region (and therefore the α4β1 integrin binding site) were prepared as described previously [17].

### Integrin constructs

Construction of pLXIN (Clontech) retroviral vectors encoding α4 integrin chimeras was previously described [17]. α4 cytoplasmic domain mutations were introduced in the full length α4 chain by site-directed mutagenesis using the QuickChange kit (Stratagene). Alanine substitutions were introduced at Y991 and E983 using the following primers: 5'-C AGA AGA GAC AGT TGG AGT GCT ATC AAC AGT AAA AGC AAT G-3' (sense) and the corresponding antisense primer for substitution of Y991 (Y991A), and 5'-C AAA TCT ATC CTA CAA GAA GCA AAC AGA AGA GAC AGT TGG-3' (sense) with the matching antisense for substitution of E983 (E983A). Double mutants were generated by successive rounds of mutagenesis, first E983, followed by Y991 substitution. Alanine and aspartic acid substitutions were introduced at S988 and S990 using the following sense primers: S988A, 5'-C CTA CAA GAA GAA AAC AGA AGA GAC GCT TGG AGT TAT ATC AAC AG-3'; S988D, 5'-C CTA CAA GAA GAA AAC AGA AGA GAC GAT TGG AGT TAT ATC AAC AG-3'; S990A, 5'-GAA AAC AGA GAC AGT TGG GCT TAT ATC AAC AGT AAA AGC-3'. Base substitution at the sites of mutation was confirmed by DNA sequencing. The nomenclature used for the different constructs is as follows: α4Y991A, α4E983A, α4EY, α4S988A, α4S988D, α4S990A.

### Paxillin constructs

The cDNA sequence encoding wild-type chicken paxillin, and paxillin with three Y to F substitutions at Y31, Y118 and Y 182, were a gift from I Dikic (Uppsala, Sweden). PCR was used to generate a *Xba I *site replacing the ATG of paxillin at the 5' end, and a *Xho I *site at the 3'end. A *Not I-Xba I *linker fragment including a *Xho I *site and a FLAG tag, DYKDDDDK, was ligated at the 5' end of the paxillin sequence. The sequence encoding the FLAG tag in frame with paxillin was excised using *Xho I *and cloned into the pMSCVpuro retroviral vector (Clontech). Paxillin lacking the LD4 domain was generated by two sequential rounds of site-directed mutagenesis, using the following deletion primers: 5'-GCT TCT TCA GCT ACA CGA GAA GAG CTG ATG GCG TCC CTC-3' and 5'-GAG GGA CGC CAT CTC TTC TCG TGT AGC TGA AGA AGC-3'. All cDNAs were sequenced.

### Cell culture

COS7, NIH 3T3 and GP+86 cells were grown in Dulbecco's modified Eagle's medium (DMEM) supplemented with 10% fetal calf serum (FCS), penicillin/streptomycin, and 2 mM glutamine (Sigma). Rat pheochromocytoma (PC12) cells were grown on Poly-D-Lysine-coated tissue culture flasks in DMEM supplemented with 10% horse serum (HS), 5% FCS, penicillin/streptomycin, and 2 mM glutamine. For priming with NGF, PC12 cells were passaged onto collagen-coated (calf-skin type I collagen; Sigma) dishes at a density of 10^4 ^cells/cm^2 ^and cultured for 4–5 days in DMEM complemented with 1% ITS^+™ ^premix (BD Biosciences) and 50 ng/ml 2.5S NGF (Serotec). For neurite outgrowth assays, NGF-primed cells were passaged by gentle trituration and cultured on the desired substrate in the same defined medium.

### Transfection and selection of stable clones

The constructs were transfected into the GP+86 packaging cell line, using FuGENE 6 as transfection reagent (Roche). Cells were maintained in DMEM supplemented with 10% FCS, penicillin/streptomycin, 2 mM glutamine and 1 mg/ml G418 (Life Technologies) for pLXIN construct or 2 μg/ml puromycin for pMSCV constructs until clones appeared. The GP+86 cells were then replated and when at 60–70% confluence, were washed and medium without G418 or puromycin added. The conditioned medium was collected after 24 h, filtered (0.45 μm pore size), and either used immediately or aliquoted, frozen and kept at -70°C. Virus titer was determined using NIH 3T3 cells and equalized. PC12 cells were transfected using polybrene, and selected and maintained with 1 mg/ml G418 or 2 μg/ml puromycin.

### Neurite outgrowth assay

All neurite outgrowth assays were performed in 4-well tissue culture plates. For preparation of the substrates, ligands (recombinant Fn fragments, type-1 collagen (Sigma) or laminin-1 (Sigma)) were diluted into PBS, and deposited as 30 *μ*l drops in the centre of the wells. Where recombinant Fn fragments were used, the dishes were first coated with 10 *μ*g/ml anti-IgG_1 _Fc for 3 h at RT, washed 2 × with PBS and then post-adsorbed with the various fragments overnight at 4°C. The wells were washed 3 × with PBS, and PC12 cells were deposited in 30 *μ*l drops on the substrates at a density of 5 × 10^2^/cm^2^. After 1 h, when the cells were attached, the medium was added, to a final volume of 200 *μ*l. Measures were taken after 12 h, and 24 h (separate sets of dishes). Dishes were viewed under a Zeiss phase microscope and random fields were recorded using a digital camera and analyzed using the Openlab software (Improvision). The length of neurites was determined using the Openlab software. Determinations were made on at least 5 separate experiments, and the longest neurite of at least 100 PC12 cells/well was measured. Untransfected and mock-transfected cells were used as negative controls. Assays were conducted on both experimental and control cell lines in parallel, and the results normalized to the control values. Each assay was repeated 5 times with three independently generated cell lines, and the mean determined. Statistical analysis was performed using Students t test.

### Immunoprecipitations and Western blotting

Cell surface molecules were labelled with 0.1 mg/ml NHS-LC-Biotin (Pierce) in PBS at 37°C for 30 min. Cells were washed three times with cell wash buffer (50 mM Tris-HCl pH 7.5, 0.15 M NaCl, 1 mM CaCl_2_, 1 mM MgCl_2_), scraped, and lysed on ice for 30 min in extraction buffer: cell wash buffer plus 1% Triton X-100, 0.05% Tween 20, 2 mM PMSF, 1 *μ*g/ml pepstatin A, 2 *μ*g/ml aprotinin, 5 *μ*g/ml leupeptin, 2 mM sodium vanadate, 4 mM sodium pyrophospate, and 2 mM sodium fluoride. After clarification by centrifuging at 14000 rpm for 20 min at 4°C, the cell lysates were incubated with 30 *μ*l protein A-sepharose (Pharmacia). The lysates were pre-cleared by two sequential 2 h incubations (to remove proteins showing non-specific interactions with protein-A) and immunoprecipitations were then carried out overnight at 4°C. Where hamster or mouse monoclonal antibodies were used, rabbit anti-hamster or rabbit anti-mouse antisera (Nordic Immunological Laboratories) were also added to the tubes (1 in 250). All antibodies were used at a dilution of 1:250. The beads were washed 5 × with immunoprecipitation buffer (identical to cell wash buffer except for 0.5 M NaCl and 1% NP40) and the precipitated polypeptides were extracted with SDS sample buffer. Precipitated cell surface biotin-labelled molecules were separated by SDS-PAGE under non-reducing conditions and detected with streptavidin-peroxidase followed by ECL (Amersham). Co-precipitated paxillin was detected by immuno-blotting of reduced immunoprecipitates with anti-paxillin antibody, followed by anti-mouse-peroxidase conjugated antibody and ECL.

## Authors' contributions

MV performed all the experiments in this study, with UBF contributing to those analysing the effects of paxillin over-expression. JH generated and validated the phospho-specific anti-α4 integrin antibody. Cff-C and MHG conceived the study, based on previous work in their respective laboratories. Cff-C wrote the first draft of the manuscript, with subsequent contributions from all authors who also approved the final manuscript.
